# Preoperative Anxiety Management Practices in Pediatric Anesthesia: Comparative Analysis of an Online Survey Presented to Experts and Social Media Users

**DOI:** 10.2196/64561

**Published:** 2025-01-27

**Authors:** Armin Sablewski, Christine Eimer, Marcus Nemeth, Clemens Miller

**Affiliations:** 1 Department of Anesthesiology and Intensive Care Medicine, University Medical Center Schleswig-Holstein, Campus Kiel, Arnold-Heller-Straße 3, Haus R3, Kiel, 24105, Germany, +49 431 500 20701; 2 Department of Anesthesiology, University Medical Center Goettingen, Goettingen, Germany; 3 Department of Anesthesiology, Children’s Orthopedic Hospital Aschau im Chiemgau, Aschau im Chiemgau, Germany

**Keywords:** pediatric anesthesia, pharmacological interventions, nonpharmacological interventions, preoperative, anxiety, anxiety management, practices, anesthesia, comparative analysis, online survey, preoperative anxiety, challenges, postoperative outcome, pediatric, infant, baby, neonatal, toddler, child, social media, survey, anesthesia provider

## Abstract

**Background:**

Managing preoperative anxiety in pediatric anesthesia is challenging, as it impacts patient cooperation and postoperative outcomes. Both pharmacological and nonpharmacological interventions are used to reduce children’s anxiety levels. However, the optimal approach remains debated, with evidence-based guidelines still lacking. Health care professionals using social media as a source of medical expertise may offer insights into their management approaches.

**Objective:**

A public survey targeting health care professionals was disseminated via social media platforms to evaluate current practices in anxiety management in children. The same questions were posed during an annual meeting of pediatric anesthesiologists with their responses serving as reference. The primary objective was to compare pediatric anesthesia expertise between the groups, while secondary objectives focused on identifying similarities and differences in preoperative anxiety management strategies hypothesizing expertise differences between the groups.

**Methods:**

Two surveys were conducted. The first survey targeted 100 attendees of the German Scientific Working Group on Pediatric Anesthesia in June 2023 forming the “Expert Group” (EG). The second open survey was disseminated on social media using a snowball sampling approach, targeting followers of a pediatric anesthesia platform to form the “Social Media Group” (SG). The answers to the 24 questions were compared and statistically analyzed. Questions were grouped into 5 categories (pediatric anesthesia expertise, representativity, structural conditions, practices of pharmacological management, and practices in nonpharmacological management).

**Results:**

A total of 194 responses were analyzed (82 in EG and 112 in SG). The EG cohort exhibited significantly greater professional experience in pediatric anesthesia than the SG cohort (median 19 vs 10 y, *P*<.001), higher specialist status (97.6% vs 64.6%, *P*<.001), and a greater pediatric anesthesia volume (43.9% vs 12% with more than 500 cases per year, *P*<.001). Regarding the representativity, 2 items out of 4 were statistically significant (level of care of institution, annual caseload of institution). Regarding the overall anxiety management practices used, there is a heterogeneous response pattern within both groups.

**Conclusions:**

Despite heterogeneous approaches, health care professionals using social media demonstrated less expertise in pediatric anesthesia but showed minimal differences in the daily management of preoperative anxiety compared with pediatric anesthesia experts. Our study highlights the potential for meaningful use of social media but future studies should explore the impact of social media health care professionals’ knowledge in other specific topics. Additionally, regarding preoperative anxiety, further recommendations are needed that could help to standardize and improve anxiety levels in children.

## Introduction

Although “no fear” is the first of the “10-N quality criteria” in pediatric anesthesia, preoperative anxiety remains prevalent [1,2]. It is evident that high levels of anxiety are associated with decreased cooperativeness during induction of anesthesia, increased postoperative analgesic requirements, increased rates of postoperative delirium, and maladaptive behavioral problems [3,4]. Therefore, it is crucial to keep anxiety levels low.

Established options for preoperative anxiolysis in children include pharmacological and nonpharmacological interventions. Midazolam, clonidine, and dexmedetomidine as well as (s-)ketamine are frequently used for pharmacological premedication [5,6]. However, the general use of these drugs is subject to controversial debate [6,7]. Nonpharmacological interventions include parental presence at induction of anesthesia, educational approaches (eg, informational mediation and prior inspection of the operating room), complementary medical procedures (such as acupuncture, music therapy, hypnosis), and cognitive-behavioral therapeutic measures (such as strengthening coping strategies, distraction, breathing exercises, model learning) [8-14]. Nonpharmacological interventions have been shown to be at least as effective as the administration of midazolam [15]. Although many different options are available, there are currently no evidence-based recommendations and guidelines on which intervention is best for which situation. Recently, we conducted a survey on the current practice of preoperative anxiety management in pediatric anesthesia among German-speaking participants [16]. It was conducted during an expert meeting of the Scientific Working Group for Pediatric Anesthesia of the German Society of Anesthesiology and Intensive Care Medicine and revealed relevant differences in the structural conditions, management of pharmacologic premedication, and the use of nonpharmacologic measures [16].

However, participants in expert meetings may not accurately reflect the realities of daily anxiety management practices. Social media, defined as “any form of electronic communication [...] to share information” [17], offers the potential to enhance these insights by leveraging swarm intelligence and engaging a broader and more diverse group of health care professionals involved in preoperative anxiety management. Web-based surveys disseminated through social media targeting pediatric anesthesia health care professionals could thus capture a larger, geographically diverse sample, enhancing overall insight. While web-based surveys are efficient and cost-effective, they have limitations, such as open participation, low response rates (≈10%), and uncertain respondent identity [18]. In contrast, closed-group surveys, such as those conducted among expert meeting participants, provide more defined and reliable profiles, potentially serving as a reference for comparison.

Thus, a web-based survey on preoperative anxiety was sent to social media users involved in pediatric anesthesia, and their responses were compared with those from a pediatric anesthesia Expert Group (EG). The study aimed to test the hypothesis that expertise and preoperative anxiety management practice differ between a broad, randomly selected social media population and a dedicated EG.

## Methods

### Overview

The web-based survey was aimed at anesthesiologists who are active on social media. Currently, there are approximately 27,000 anesthesiologists in Germany, around 3300 in Austria, and 1600 in Switzerland [19-21]. It is estimated that approximately 70%‐90% of all physicians actively use social media, meaning that around 25,000 anesthesiologists were eligible to participate in the web-based questionnaire [22,23]. As this was an open survey, the participation of nonanesthesiologists could not be excluded. The survey related to the scientific conference was directed at the scientific working group on pediatric anesthesia in German-speaking countries. It consists of physicians with predominantly high expertise in the field of pediatric anesthesia, with approximately 100 participants attending the conference each year.

### Ethical Considerations

This analysis did not require approval by an institutional review board or entry into a clinical trial register since it did not include data from patients or medical records according to the Helsinki Declaration. Participation was voluntary, and privacy was ensured through the anonymous collection of study data. No personal information, cookies, or IP addresses that could enable identification were stored. Participants did not receive any compensation.

### Survey Development

We adhered to the items of the “Good practice in the conduct and reporting of survey research” and the Checklist for Reporting Results of Internet E-Surveys (CHERRIES) [24,25]. Technical tests were carried out before the surveys were conducted to enhance comprehensibility and rule out possible errors. The completion rate (ratio of users who finished the survey/users who answered the first question) was calculated for both groups and the participant rate (ratio of unique visitors who agreed to participate/unique first survey page visitors) for EG. For completeness checking, incomplete data were marked as “not applicable” to indicate the extent of survey completion. The processed data consisted of 2 surveys, each conducted independently. There were no follow-up validation attempts to verify if the respondents were truly qualified.

### Data Sources

The first survey was conducted among participants of the annual meeting of the Scientific Working Group on Pediatric Anesthesia of the German Society for Anesthesiology and Intensive Care medicine (DGAI), which took place in Hamburg, Germany, on June 16‐17, 2023. During the event, access to a web-based survey, using Microsoft Forms (Office 365), was given via a QR code. The survey contained 25 questions targeting the daily practice of preoperative anxiety management in children (Multimedia Appendix 1). Those respondents formed the EG, serving as a reference to the second survey. The results of this survey were formerly published [16].

The second open survey was announced among followers of a German-language podcast on pediatric anesthesia [26]. This podcast is broadcast on platforms such as Spotify and Apple Podcast (in total 44 platforms) and has achieved approximately 130k downloads and streams with its 34 episodes (data retrieved on July 01, 2024). Users were given access to this survey from October 01 to 31, 2023. The first call for participation was made on October 01, 2023, via short posts on the social media platforms X, Bluesky, and Instagram, as well as posts on the corresponding social media accounts in the field of anesthesiology following the random snowball sampling method. The invitation included an image with a QR code and a link to the web-based survey (Multimedia Appendix 2) along with a request for reposts. Several reposts were made and a short podcast episode on October 19, 2023, was broadcast to recall for participation (1117 downloads in the survey period). The episode was available on major podcast platforms, including Podigee, Spotify, Apple Podcasts, Amazon Podcasts, and Google Podcasts [27]. In comparison to the first survey, this one was expanded to cover the broad spectrum of social media users with 4 more questions (marked with asterisk (*) in Multimedia Appendix 1). Respondents formed the “Social Media Group” (SG).

All items were displayed on a website and were only interrupted by adaptive questions. Completeness checks before submission were not integrated, and respondents were able to modify their answers before submission. For both surveys, multiple participation could not be technically excluded but respondents of the second survey were asked to refuse participation in case of prior participation to the first survey. Multiple selections were possible for some questions.

### Data Processing

Both survey data were checked for incomplete data and then matched using Microsoft Excel (Office 2019, Microsoft). The questions were clustered into five categories: (1) pediatric anesthesia expertise (3 items), (2) representativity (5 items for both surveys and 4 additional items in SG), (3) structural conditions (9 items), (4) practices of pharmacological routine (6 items), and (5) practices of nonpharmacological routine (2 items). The item “zip codes” and the 4 additional questions were excluded from the comparison between the 2 groups, resulting in a total of 24 items being compared.

### Objectives

The primary objective was to assess the pediatric anesthesia expertise of the SG compared with the EG, given by significant differences in professional expertise (defined by years of professional experience), expert status (defined as being a board-certified anesthesiologist with passed professional examination), and a number of personal annual pediatric anesthesia case volume. The secondary objectives were the differences in the clustered categories of general characteristics and practices in managing preoperative anxiety, capturing structural conditions and practices in both pharmacological and nonpharmacological interventions.

### Statistical Analysis

In the descriptive analysis, we presented the absolute and relative frequencies for the respective groups for categorical variables and the medians, IQRs, and total ranges for the respective groups for continuous variables. We applied a significance level of 0.05 for all statistical tests. The Kolmogorov-Smirnov test was used to assess the normality of the distribution. *P* values for the comparison of both groups were calculated using Fisher exact test or chi-square test for categorical variables, and the Wilcoxon rank sum test for continuous variables. Analysis and illustrations were performed using GraphPad Prism (GraphPad Software) and Microsoft Excel (Office 2019, Microsoft).

## Results

### Overview

**Figure 1.**
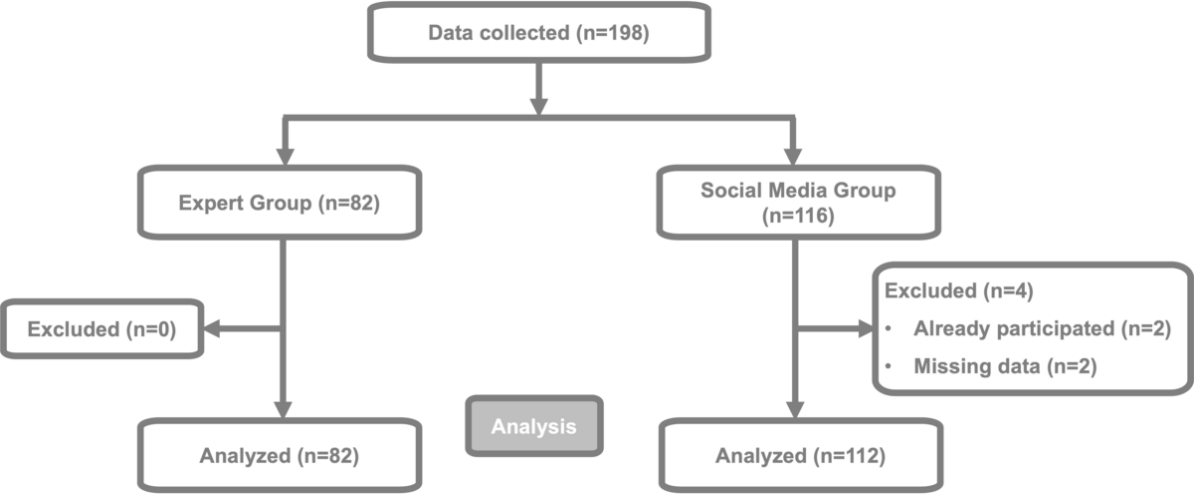
Flow chart of data collection and analysis.

A total of 198 respondents participated in both surveys, 82 respondents in the EG and 116 respondents in the SG, respectively. Two responses from the SG were excluded due to prior participation in the EG survey, and another 2 responses were excluded due to missing data, leaving 194 responses for the final analysis in both groups (Figure 1). Unless stated otherwise, the full analysis set consisted of 82 respondents in the EG and 112 in the SG, with nonrespondents excluded from all calculations.

The participation rate in EG was 82% as 82 participants out of 100 joined the survey during the annual meeting. Since no IP addresses were recorded, the participation rate for the SG could not be calculated. The completion rate of analyzed data was 100% in both groups.

In the 5 clustered categories, we found 10 out of 24 items to be significantly different in the response behavior between the 2 groups (see Table 1, corresponding questions in Multimedia Appendix 1).

**Table 1.** Survey items in clustered categories with differences between the Expert Group (EG) and the Social Media Group (SG). Detailed evaluation in the text.

Category and item		*P* value
Pediatric anesthesia expertise		
	Years of professional experience	<.001
	Specialist (board-certified anesthesiologist)	<.001
	Personal pediatric anesthesia case volume annually	<.001
Representativity		
	Gender	.51
	Country of respondents’ institution	.65
	Level of care of the institution	<.001
	Institutional pediatric anesthesia case volume annually	<.001
Structural conditions		
	Written protocols for managing preoperative anxiety	.47
	Existing preoperative preparation programs	.76
	Used preoperative preparation programs	.90
	Feasibility (local conditions) of parental presence during induction of anesthesia	.01
	Standard of parental presence during induction of anesthesia	.69
	Place of separation of the children from their caregivers	.84
	Routine in anxiety measurement	.16
	Used anxiety measurement tools	—^a^
	Known anxiety measurement tools	<.001
Practices of pharmacological management		
	Regular use of preoperative medication	.99
	Indication-based prescription of premedication, avoiding routine use	.04
	Criteria for deciding on premedication use	.24
	Most commonly used substance	.23
	1st choice for premedication	.999
	Minimum age for administering premedication	<.001
Practices of nonpharmacological management		
	Standard practice of nonpharmacological interventions	.63
	Use of nonpharmacological interventions	<.001

a Not applicable (only 4 responses in the EG and only 1 response in the SG, no statistical analysis was carried out).

### Pediatric Anesthesia Expertise

The level of pediatric anesthesia expertise demonstrated by the SG was significantly lower than that reported in the EG. This was given by a lower number of professional experience in the SG with a median of 10 years (IQR 6‐18; total range 1‐45) compared with a median of 19 years (14-25; 5-35) in the EG (*P*<.001), a lower share of respondents in the SG group of specialists, with 64.6% (64/99, with 13 nonrespondents), compared to 97.6% (80/82) in the EG group (*P*<.001), a lower share of respondents in the SG group reported performing more than 500 pediatric anesthesia cases per year, with 12% (13/108, with 4 nonrespondents), compared to 43.9% (36/82) in the EG group (*P*<0.001; details in Table 2).

**Table 2.** Distribution of performed anesthesia case volume per year.

Personal pediatric anesthesia case volume annually	EG^a^ (n=82), n (%)	SG^b^ (n=108)^c^, n (%)
0‐49	4 (4.9)	50 (46.3)
50‐99	1 (1.2)	17 (15.7)
100‐199	13 (15.9)	15 (13.9)
200‐299	11 (13.4)	7 (6.5)
300‐399	11 (13.4)	5 (4.6)
400‐499	6 (7.3)	1 (0.9)
>500	36 (43.9)	13 (12)

a EG: Expert Group.

b SG: Social Media Group.

c 4 nonrespondents; total n=112.

### Representativity

Both groups showed a similar gender distribution, with 56.6% (45/81, 1 nonrespondent) female respondents in EG compared to 50% (56/112) in SG (*P*=.51) and the same percentage of respondents from Germany (93.8% each; 76/81, with 1 nonrespondent, in EG and 105/112 in SG). In the EG, respondents originated from 3 more countries (Switzerland, Austria, and Italy), whereas in the SG, respondents came from 5 more countries (Switzerland, Austria, Serbia, United Kingdom, and Hungary).

Differences in the level of care across respondents’ workplaces were statistically significant (*P*<.001, Table 3). Most respondents came from university hospitals (28/81, 1 nonrespondent, 34.6% in EG vs 33/112, 29.5% in SG) while more respondents came from standard care hospitals in the SG (27/112, 24.1%) than in the EG (6/81, 1 nonrespondent, 7.4%).

**Table 3.** Level of care across respondents’ workplaces.

	EG^a^ (n=81)^b^, n (%)	SG^c^ (n=112), n (%)
Ambulatory	6 (7.4)	10 (8.9)
Standard care hospital	6 (7.4)	27 (24.1)
Children’s hospital	18 (22.2)	11 (9.8)
High care hospital	17 (21)	27 (24.1)
University hospital	28 (34.6)	33 (29.5)
Others	6 (7.4)	4 (3.6)

a EG: Expert Group.

b 1 nonrespondent; total n=82.

c SG: Social Media Group.

The annual pediatric anesthesia caseloads varied between groups. In the EG group, most 71.3% (n=57) reported over 1000 cases annually, with smaller proportions handling 500-999 (n=11, 13.8%), 250-499 (n=10, 12.5%), or fewer than 250 cases (n=2, 2.5%). In the SG group, 34.6% (n=36) managed over 1000 cases, 22.1% (n=23) reported 500-999, 18.3% (n=19) had 250-499, and 25% (n=26) handled fewer than 250 cases. Some respondents in both groups did not answer (n=2 in EG and n=8 in SG).

### Structural Conditions

In EG, 36 (43.9%) respondents reported having a written standard operating procedure for managing preoperative anxiety, compared with 43 (38.4%) respondents in SG (*P*=.46). A preoperative preparation for children and their caregivers was included as part of anesthesia information by 28 (34.1%) respondents in EG and by 35 (31.3%) respondents in SG (*P*=.76). Among those who reported using specific material, there was no difference in the choice of measures (*P*=.90). The most frequently used materials were “pediatric-specific informed consent”, information flyers, and comics (Table 4).

**Table 4.** Materials used to help prepare children and their caregivers preoperatively.

	EG^a^ (n=28), n (%)	SG^b^ (n=35), n (%)
Pediatric-specific informed consent	19 (25.5)	23 (20.5)
Information flyer	16 (21.5)	15 (13.4)
Comics	8 (10.7)	10 (8.9)
A designed mascot	7 (9.4)	8 (7.1)
Videos	4 (5.4)	2 (1.8)
Other^c^	2 (2.7)	3 (2.7)
Hypnosis	1 (1.3)	0 (0)
Guidance through the operating room	2 (2.7)	1 (0.9)

a EG: Expert Group.

b SG: Social Media Group.

c Other used materials mentioned: instruction on how to use topical anesthesia patches, offering website information, the use of soap bubbles, and a virtual operating theater tour.

When asked whether the local structural conditions would generally allow the parents to be present until the children are anesthetized, 52 (63.4%) respondents in the EG and 50 (45%) respondents in the SG answered in the affirmative (*P*=.01). Among those, 38 (46.3%) respondents in the EG and 57 (50.9%) respondents in the SG reported not offering parents to be present during the induction of anesthesia. In EG, 26 (31.7%) respondents reported enabling parental presence while 29 in SG (25,9%). Another 18 (22%) respondents in the EG stated that parental presence depends on the individual workplace within their institution, while 26 (23.2%) respondents in the SG reported the same. The place where the children were separated from their parents or parental substitutes did not differ significantly between the groups.

Separation locations for children from parents did not significantly differ between groups (*P*=.84). Most commonly, separation occurred during transfer to the operating room (EG: 44 out of 80 respondents, 55%; SG: 67 out of 109 respondents, 61.5%). Separation in the induction room was less frequent (EG: 13 out of 80 respondents, 16.3%; SG: 13 out of 109 respondents, 11.9%) and in the holding area similarly rare (EG: 12 out of 80 respondents, 15%; SG: 13 out of 109 respondents, 11.9%). Separation in the operating room itself was reported even less often (EG: 9 out of 80 respondents, 11.3%; SG: 12 out of 109 respondents, 11%), while on the ward it was rarest of all (EG: 2 out of 80 respondents, 2.5%; SG: 4 out of 109 respondents, 3.7%). A small number of respondents did not provide an answer (EG: 2 respondents; SG: 3 respondents).

A total of 95.1% (78/82) of respondents in EG and 99.1% (111/112) in SG reported that children’s anxiety is not routinely measured. Regarding anxiety scales, 31.7% (n=33) of EG respondents and 55.8% (n=67) of SG respondents stated that they were not familiar with any. The Yale Preoperative Anxiety Scale was the most recognized scale in the EG, with 25% (n=26) of respondents indicating familiarity with it. In contrast, only 8.3% (n=10) of respondents in the SG reported familiarity with the Yale Preoperative Anxiety Scale. The visual analog scale (VAS) was the most recognized scale in the SG, with 30% (n=36) of respondents indicating familiarity with it. In contrast, only 24% (n=25) of respondents in the EG reported familiarity with the VAS.

### Practices of Pharmacological Interventions

The use of pharmacological premedication in daily practice was reported by 80.5% of respondents (66/82) in EG and 79.5% (89/112) in SG, with no statistically significant difference between the 2 groups (*P*>.99), indicating that both groups have a similarly high rate of routine use of premedication.

When it comes to actively avoiding premedication, there was a significant difference between the 2 groups (*P*=.04). In EG, 50% (41/82) tried to avoid premedication, whereas only 34.4% (39/112) in the SG did so.

Both groups showed similar responses (*P*=.24) regarding their decision-making process for administering pharmacologic premedication (refer to Table 5). Individual responses included consulting children or their parents about the need for premedication, with some also specifying the placement of an intravenous line before anesthesia induction.

**Table 5.** Criteria for deciding on premedication use. Multiple answers were possible.

	EG^a^ (n=82), n (%)	SG^b^ (n=112), n (%)
The children are generally premedicated with medication	28 (34.1)	55 (49.1)
According to the child’s anxiety	52 (63.4)	52 (46.4)
According to the parents’ anxiety	18 (22)	18 (16.1)
According to the child’s wishes	38 (46.3	35 (31.3)
According to the parents’ wishes	22 (26.8)	25 (22.3)
According to medical history	46 (56.1)	52 (46.4
According to experience/gut feeling	31 (37.8)	46 (41.1)
Individual answer	12 (14.6)	10 (8.9)

a EG: Expert Group.

b SG: Social Media Group.

In both groups, midazolam was reported as the most frequently used premedication drug (EG: 81/82, 98.8% and SG: 112/112, 100%). In EG, (es-)ketamine (41/82, 50%), clonidine (18/82, 22%), and dexmedetomidine (6/82, 7.3%) were used as well as in SG (42/112, 37.5%; 19/112, 17%; and 11/112, 9.8%, respectively) without significant difference (*P*=.23). Overall, midazolam was the drug of first choice in both groups (76/80, 2 nonrespondents, 95% in EG vs 105/111, 1 nonrespondent, 94.6% in SG).

The median minimum age for administering premedication was 6 months (6-8; 0-48) in EG and 9.5 months (6-12; 0-36) in SG (*P*<.001).

### Practices of Nonpharmacological Interventions

Nonpharmacological interventions were routinely used by 60 (73.2%) respondents in EG and by 78 (69.6%) respondents in SG (*P*=.63). There was a significant difference (*P*<.001) in the selection of which nonpharmacological interventions were used (Table 6). While in the EG parental presence was the most reported intervention (43/60, 71.7%), it was the use of videos in the SG (62/78, 79.5%).

**Table 6.** Practices of nonpharmacological interventions. Multiple answers were possible.

	EG^a^ (n=60), n (%)	SG^b^ (n=78), n (%)
Parental presence	43 (71.7)	38 (48.7)
Videos (tablet, smartphone, etc)	42 (70)	62 (79.5)
Reading or showing books	21 (35)	29 (37.2)
Games	17 (28.3)	14 (17.9)
Other activities^c^	12 (20)	12 (15.4)
Audio books	8 (13.3)	5 (6.4)
Music distraction	7 (11.7)	14 (17.9)
Hypnosis	7 (11.7)	2 (2.6)
Behavioral exercises	7 (11.7)	0 (0)
Clowns	5 (8.3)	4 (5.1)
Virtual reality glasses	2 (3.3)	4 (5.1)

a EG: Expert Group.

b SG: Social Media Group.

c Other activities mentioned in both groups were the use of a floating bird, the integration of cuddly toys, the use of a glitter wand, interactive storytelling, a starry sky projection, and the use of soap bubbles.

## Discussion

### Principal Results

The respondents to the publicly announced survey on social media demonstrated significantly less pediatric anesthesia expertise than the respondents to the survey among experts. This was evidenced by fewer years of professional experience, fewer board-certified specialists, and a lower pediatric anesthesia caseload. However, when looking at the items related to the practice of pediatric anxiety management, significant differences were found in less than a third. Regardless of the survey group, our results showed very heterogeneous approaches to the management of preoperative anxiety in pediatric patients.

Our study presents 2 principal findings. First, it remains debatable whether web-based surveys are an effective method for reaching the target group of pediatric anesthesia providers. On the one hand, the respondents to the web-based survey rated their level of expertise lower than those who were involved in a scientific meeting survey. However, specific parameters that best identify an expert in the field of pediatric anesthesia remain undefined. It seems clear that increased experience in this field correlates with a lower rate of complications in children [28]. A high volume of pediatric anesthesia cases likely contributes to a higher level of expertise. Being classified as a specialist (board-certified anesthesiologist) further indicates that a minimum standard of experience in pediatric anesthesia has been met [29]. However having many years of professional experience does not necessarily equate to extensive pediatric anesthesia practice, as hospital structure, hospital focus, and patient demographics may limit exposure to pediatric cases [30,31]. In addition, the higher share of institutions with a higher level of care and a higher number of children’s hospitals among experts might indicate pediatric anesthesia expertise due to a higher pediatric caseload. But there may be also anesthesia providers with a high individual pediatric caseload in standard care hospitals. On the other hand, when responses from an EG exhibit significant heterogeneity [16], it is not unexpected that similar heterogeneity would persist when querying a larger (or another) sample. This leads to the conclusion that it is not that obvious as our results may indicate which of the 2 groups can more accurately reflect the actual reality of pediatric anesthesia care.

Further, the dissemination of a web-based survey through social media is debatable. The methodology of web-based surveys offers significant advantages, particularly due to their rapid deployment and extensive reach, which facilitate the swift collection and distribution of data. Similarly, social media, which has become increasingly popular in the medical field, enables the rapid dissemination of insightful opinions and information and underscores the value of web-based surveys [23,32]. Drawbacks of web-based surveys are the inadequate representation of the sample population due to insufficient coverage, the absence of a sampling frame to guide sample selection, nonselection bias, and a low participation rate, which is estimated at approximately 11% [33-35]. Of course, it is difficult to verify data quality with anonymous questions, and there is ongoing research into how to implement attention checks or other means of detecting poor-quality data in web-based surveys [36]. The presence of selection bias within the SG is also possible. This could skew the data, as operating within a “bubble” may predominantly reach individuals already familiar with the topic, potentially also limiting a full representation of reality. An interesting direction for future research could involve comparing groups with similar characteristics to examine whether the survey access method (social media vs conference or “classic”) introduces a selection effect related to expertise. With a participation rate of more than 80%, the EG directly addressed at the conference meeting demonstrated a high willingness to participate. Overall, slightly more responses were collected in the SG, even though access was open for an extended period and potentially a higher amount of respondents, indicating a low response rate in this “digital” SG. This suggests that if a survey on a specific topic, such as anxiety in pediatric anesthesia, is announced via social media, it is likely that only a specific subset of individuals, those with a particular interest or relevance to the topic, will actively engage and participate.

The second point, apart from the discussion about whom to ask for pediatric anesthesia surveys, is what both groups have in common: There is a large heterogeneity in applied anxiety management practices. This includes the debated issue of parental presence during anesthesia induction. Although it does not reduce children’s anxiety, children have the right to be accompanied by their parents or substitutes. Surprisingly, parental presence during induction remains uncommon [37,38]. Local conditions appear to inhibit parental presence, and even if it was possible in principle, it is often not implemented. Potential reasons for this could include the need for additional staff or carefully coordinated arrangements to manage the logistics of parental involvement, as well as considerations pertaining to hygiene.

The same heterogeneity exists in the question of when which child should receive premedication. This is shown by the many varying approaches regardless of the 2 survey groups. Medication is currently made in a highly inconsistent manner, largely based on individual clinical judgment. When it comes to the application of medication, midazolam holds a high relevance in both groups. However, it has been proven to significantly reduce preoperative anxiety, it also has evident disadvantages including a long recovery time, respiratory adverse effects, and amnestic effects [6,7]. That may explain why all respondents reported using alternative premedication agents such as (es-)ketamine, clonidine, and dexmedetomidine frequently [5]. In addition, the application of nonpharmacological interventions is heterogeneous [15]. But if applied, one of the most favored options is video distraction. This does not seem surprising since video distraction is easy to implement, widely available, and requires no training or infrastructure (unlike, for example, clowns).

With regard to anxiety scales, it is noteworthy that 30% of the experts were unfamiliar with any scales for measuring anxiety. Awareness of specialized scales, such as the modified Yale Preoperative Anxiety Score, was higher among the EG, likely due to its frequent use in studies and the fact that many experts are affiliated with university settings [39]. In contrast, participants in the SG reported slightly greater familiarity with the VAS, a tool widely recognized for its application in pain management [40]. Despite the availability of these tools, their limited use remains puzzling. Broader implementation could enhance the identification of preoperative anxiety and increase awareness of its importance in clinical practice.

### Limitations

The study faced several limitations. First, it is prone to bias as there likely was a potential overrepresentation of more tech-savvy individuals in the SG, leading to a demographic discrepancy compared with the EG with a potential risk for self-selection bias. Second, the reach of the web-based survey could not be sufficiently quantified, and attention checks were omitted, compromising information about the response rate and its quality. The survey was disseminated through a variety of social media platforms, but without considering social media use statistics, which may have biased the sample. Additionally, the anonymity of the survey precluded verification of respondent accuracy.

### Conclusion

The respondents from a scientific working group on pediatric anesthesia had more professional experience in this medical subspecialty and also more specific knowledge than survey participants from social media. However, when it comes to the use of strategies that reflect daily practice, the groups differed little and only in general terms. A diverse range of pharmacological and nonpharmacological interventions are used in daily practice and their use seems to be based more on individual preferences. Consequently, there is a need for evidence-based recommendations regarding the appropriate use of these interventions, including indications for their use. Web-based surveys via social media can have the potential to gain insights into daily practice on specific topics like managing preoperative anxiety in pediatric patients. Further studies should investigate whether surveys disseminated through social media yield similar results in other specific subject areas.

## Supplementary material

10.2196/64561 Multimedia Appendix 1 Survey to “Preoperative Anxiety Management Practices in Pediatric Anesthesia: A Comparative Analysis of an Online Survey presented to Experts and Social Media Users.”

10.2196/64561 Multimedia Appendix 2 Image that was posted for invitation to participate to the online survey for the social media group.

## Abbreviations

CHERRIES: Checklist for Reporting Results of Internet E-Surveys
EG: Expert Group
SG: Social Media Group
VAS: visual analog scale
