# Supplementary material for: Preoperative Anxiety Management Practices in Pediatric Anesthesia: Comparative Analysis of an Online Survey Presented to Experts and Social Media Users
Source: JMIR Pediatr Parent. 2025 Jan 27;8:e64561. doi: 10.2196/64561 (PMC11790179; doi:10.2196/64561)
Supplement: Multimedia Appendix 1 [file pediatrics-v8-e64561-s001.docx]

# Multimedia Appendix 1

Survey to „Preoperative Anxiety Management Practices in Pediatric Anesthesia: A Comparative Analysis of an Online Survey presented to Experts and Social Media Users“

| I Pediatric anesthesia expertise | | | |
| --- | --- | --- | --- |
|  | Item | Question | Answer |
| 1 | Years of professional experience | How much work experience do you have? (in years rounded up) | - Open answer |
| 2 | Specialist (board-certified anesthesiologist) | Are you a medical board-certified specialist? | - Yes - No |
| 3 | Personal pediatric anesthesia case volume annually | How many pediatric anesthesia cases do you supervise per year? | - 0-50 - 50-99 - 100-199 - 200-299 - 300-399 - 400-499 - > 500 |

| II Representativity | | | |
| --- | --- | --- | --- |
|  | Item | Question | Answer |
| 1 | Gender | I am… | - Male - Female - Other |
| 2 | Country of respondents’ institution | My place of work is located in... | - Germany - Austria - Switzerland - Other (open answer) |
| 3 | Level of care of the institution | I work... | - Ambulatory - Standard Care Hospital - Children's Hospital - High Care Hospital - University Hospital - Others |
| 4 | Institutional pediatric anesthesia case volume annually | We manage approximately this many pediatric anesthesia cases per year (total number of cases) | - 0-249 - 250-499 - 500-999 - > 1000 |
| 5^a^ | Profession | I am… | - Physician - Rescue service personnel - Nursing staff - Other medical professionals - None of the above (“Other”) |
| 6^a^ | Training Specialisation | I am a training assistant in the specialty of | - Anesthesiology - Internal Medicine - Pediatrics - Surgery - Open answer |
| 7^a^ | Medical Specialisation | I am a medical specialist in | - Anesthesiology - Internal Medicine - Pediatrics - Surgery - Open answer |
| 8^a^ | Exclusion criteria | Have you already taken part in the TIGER survey at the Annual Conference 2023 in Hamburg? | - Yes - No |
| 9^b^ | ZIP Code | The postal code of my place of residence is... | - Open answer |

^a^ Questions only asked in Social Media Group

^b^The geographical locations were considered based on the ZIP codes and assessed to ensure that there are no local clusters

| III Structural Conditions | | | |
| --- | --- | --- | --- |
|  | Item | Question | Answer |
| 1 | Written protocols for managing preoerative anxiety | Does your facility have a written procedure for dealing with preoperatively anxious and/or restless children? | - Yes - No |
| 2 | Existing preoperative preparation programs | Is there a special preoperative preparation for children as part of the anesthesia information? | - Yes - No |
| 3 | Used preoperative preparation programs | If yes, what special preoperative preparation is there for children? (multiple answers possible) | - Special information for children - Information leaflet - Comics - A designed mascot - Online program/website - (Virtual) surgery tour - Videos - Open answer |
| 4 | Feasibility (local conditions) of parental presence during induction of anesthesia | Does the local conditions in your work place allow parents/guardians to accompany the children into the operating room/for anesthesia induction? | - Yes - No |
| 5 | Standard of parental presence during induction of anesthesia | Is it standard practice for parents/guardians to accompany children to the operating room/anesthesia induction? | - Yes (whenever possible) - No (only in exceptional cases) - It depends on the area - Open answer |
| 6 | Place of separation of the children from their caregivers | Where does the separation (either physically or by falling asleep) of children from their parents mainly take place?  *Sleeping room: A room (child-friendly and/or separate) before entering the operating room, in which measures for putting the child to sleep (e.g. establishment of IV access, administration of Propofol) are established | - On ward - In the holding area - In the induction room* - While transfer to operating theater - In the operating room - Other areas (open answer) |
| 7 | Routine in anxiety measurement | Is childhood anxiety routinely measured in the workplace? | - Yes - No |
| 8 | Used anxiety measurement tools | If yes: Which instrument is used? | - mYPAS - mYPAS-SF - VAS - STAI - APAIS - Other (open answer) |
| 9 | Known anxiety measurement tools | Which instrument do you know for measuring anxiety in children? | - mYPAS - mYPAS-SF - VAS - STAI - APAIS - Other (open answer) |

| IV Practices of Pharmacological interventions | | | |
| --- | --- | --- | --- |
|  | Item | Quenstion | Answer |
| 1 | Regular use of preoperative medication | Are children routinely premedicated in your workplace? | - Yes - No |
| 2 | Indication-based prescription of premedication, avoiding routine use | Is there an (active) attempt to avoid premedication with medication? | - Yes - No |
| 3 | Criteria for deciding on premedication use | How do you decide whether a child should receive premedication? (multiple answers possible) | - The children are generally premedicated with medication - According to the child's anxiety - According to the parents' anxiety - According to the child's wishes - According to the parents' wishes - According to medical history - According to experience/gut feeling - Other factors |
| 4 | Most commonly used substance | Which medication do you use?  (multiple answers possible) | - Midazolam - Clonidin - Dexmedetomidin - Ketamin/Esketamin - Other |
| 5 | 1st choice for premedication | What is the drug of first choice for premedication? | - Midazolam - Clonidin - Dexmeditomindin - Ketamin/Esketamin - Other |
| 6 | Minimum age for administering premedication | From what age are children premedicated in your facility? (in months of life) | - Open answer |

| V Practices of non-Pharmacological interventions | | | |
| --- | --- | --- | --- |
|  | Item | Question | Answer |
| 1 | Standard practice of non-pharmacological interventions | Are non-pharmacological intervention used during the induction of anesthesia? | - Yes - No |
| 2 | Use of non-pharmacological interventions | If yes, which non-pharmacological interventions are used? (multiple answers possible) | - Musical measures - Audio books (e.g. Toniebox) - Videos (tablet, smartphone...) - VR glasses - Hypnosis - Reading/showing books - Parental presence - Clowns - games - acupuncture - Behavioral exercises - Other activities (open answer) |
