# Supplementary figures and images for: Preoperative Anxiety Management Practices in Pediatric Anesthesia: Comparative Analysis of an Online Survey Presented to Experts and Social Media Users
Source: JMIR Pediatr Parent. 2025 Jan 27;8:e64561. doi: 10.2196/64561 (PMC11790179; doi:10.2196/64561)

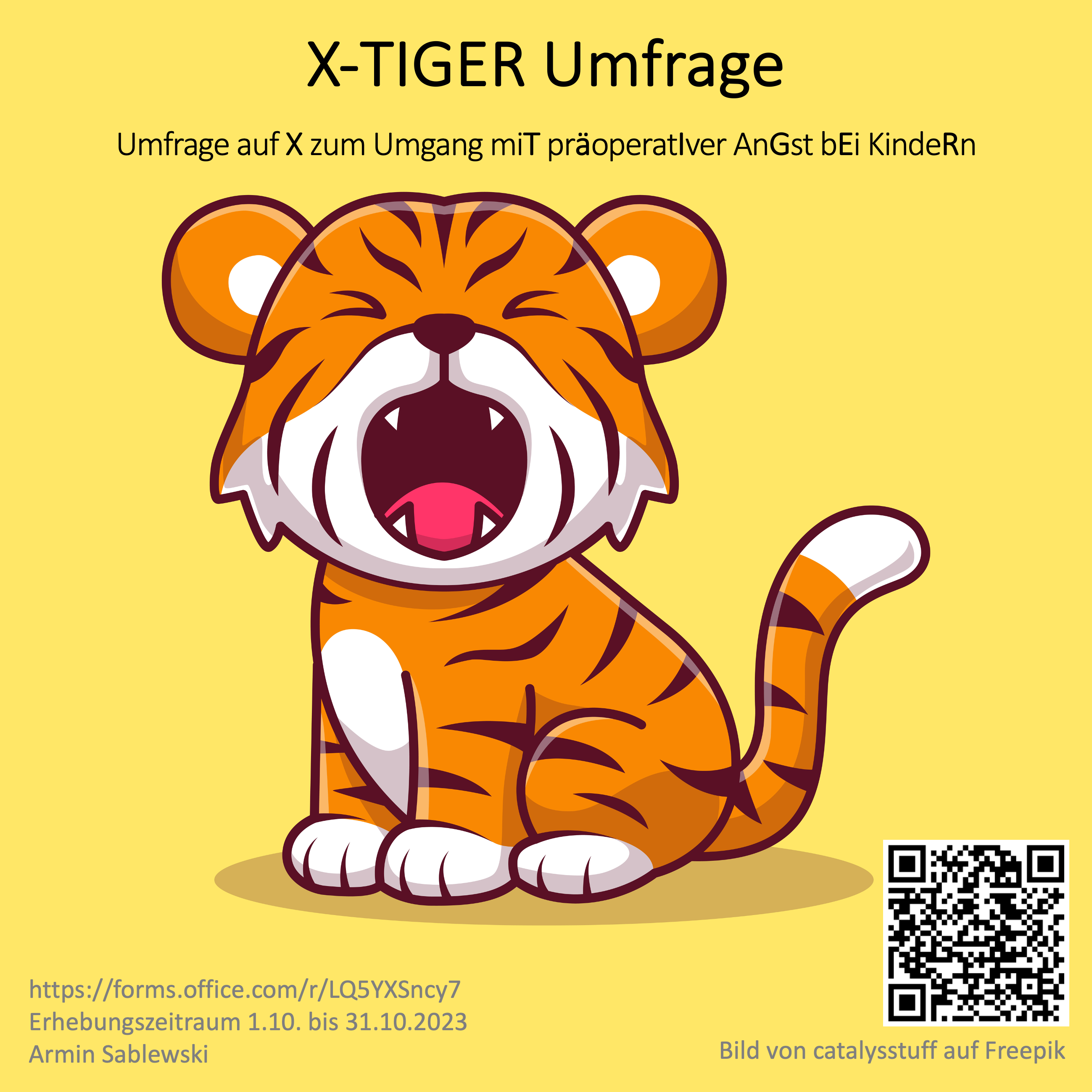

Supplement: Multimedia Appendix 2 [file pediatrics-v8-e64561-s002.png]
